# Effect of Feeding *Bacillus subtilis* Spores to Broilers Challenged with *Salmonella enterica* serovar Heidelberg Brazilian Strain UFPR1 on Performance, Immune Response, and Gut Health

**DOI:** 10.3389/fvets.2018.00013

**Published:** 2018-02-13

**Authors:** Ricardo Mitsuo Hayashi, Mariana Camargo Lourenço, Antônio Leonardo Kraieski, Raquel Bighetti Araujo, Ricardo Gonzalez-Esquerra, Eduardo Leonardecz, Anderson Ferreira da Cunha, Marcelo Falsarella Carazzolle, Paulo Sérgio Monzani, Elizabeth Santin

**Affiliations:** ^1^Laboratório de Microbiologia e Ornitopatologia, Universidade Federal do Paraná, Curitiba, Brazil; ^2^Novus International, Inc., Indaiatuba, Brazil; ^3^Laboratório de Bioquímica e Genética Aplicada, Departamento de Genética e Evolução, Centro de Ciências Biológicas e da Saúde, Universidade Federal de São Carlos, São Carlos, Brazil; ^4^Laboratório de Genômica e Expressão, Universidade de Campinas, Campinas, Brazil; ^5^Departamento de Medicina Veterinária, Faculdade de Zootecnia e Engenharia de Alimentos, Universidade de São Paulo, Pirassununga, Brazil

**Keywords:** 16S, gut health, gut microbiome, immunity, poultry, probiotic, salmonellosis

## Abstract

Salmonellosis is a poultry industry and public health concern worldwide. Recently, *Salmonella enterica* serovar Heidelberg (SH) has been reported in broilers in Brazil. The effect of feeding a blend of three strains of *Bacillus subtilis* (PRO) was studied in broilers orally challenged (10^7^ CFU/chick) or not with a SH isolated in south of Brazil (UFPR1 strain). Twelve male Cobb 500 broilers per pen were randomly assigned to six treatments in a 3 × 2 factorial experiment where PRO was added at 0, 250, or 500 g/ton of broiler feed and fed to either SH-challenged (SH Control, SH + PRO 250, and SH + PRO 500) or non-challenged birds (Control, PRO 250, and PRO 500). Broiler performance, histologic alterations in intestinal morphology, *Salmonella* quantification and immune cells counts in liver (macrophages, T CD4+ and T CD8+) were analyzed. Changes in the intestinal microbiota of broilers were also studied by metagenomics for Control, SH Control, SH + PRO 250, and SH + PRO 500 only. Feeding PRO at 250 or 500 g/ton reduced SH counts and incidence in liver and cecum at 21 days of age. It was observed that PRO groups increased the macrophage mobilization to the liver in SH-challenged birds (*P* < 0.05) but reduced these cells in the liver of non-challenged birds, showing an interesting immune cell dynamics effect. PRO at 250 g/ton did not affect gut histology, but improved animal performance (*P* < 0.05) while PRO at 500/ton did not affect animal performance but increased histologic alteration related to activation of the defense response in the ileum in SH challenged birds compared to control birds (*P* < 0.05). SH + PRO 500 group presented a more diverse cecal microbiota (Shannon–Wiener index; *P* < 0.05) compared to Control and SH Control groups; while SH + PRO 250 had greater ileal richness (JackkNife index) compared to Control (*P* < 0.05). PRO was effective in reducing *Salmonella* colonization in liver and cecum when fed at 250 or 500 g/ton to broilers inoculated with SH strain UFPR1. PRO promotes positive alterations in performance (at 250 g/ton), immune modulatory effect in the gastrointestinal tract, SH reduction, and intestinal microbiota modulation.

## Introduction

Despite advances in the treatment of infectious diseases, pathogenic microorganisms such as *Salmonella* are an important threat to both human and animal health worldwide (1). *Salmonella* is a pathogen but it also has the ability to live in animals and poultry as a transient member of the intestinal microbial population without causing disease. Colonization of most types of *Salmonella enterica* does not often affect poultry performance and consequently asymptomatic infections may increase the likelihood of zoonotic transmission to humans through the food chain (2). *S. enterica* serovar Heidelberg (SH) ranks among the top three serovars isolated from patients with salmonellosis in North America, higher than in other regions of the world (3), provoking more invasive infections (e.g., myocarditis and bacteremia) than others non-typhoidal *Salmonella* (4). The Brazilian SH strain used in this trial (UFPR1) had its complete genome described recently, showing high resistance to short-chain organic acids and intermediate resistance to some antibiotics (5).

Oral administration of probiotics may reduce the intestinal colonization of *Salmonella* (6, 7), along with the inflammation caused by this bacterium, in broiler chickens (8). Probiotics are live microorganisms that offer an advantage to their hosts by enhancing the hosts’ beneficial microbiota (9, 10). Studies have demonstrated that *Bacillus* spp. and *Bacillus subtilis* spores may be successful competitive exclusion agents (11). *B. subtilis* modulates the intestinal microbiota and favors the growth of lactic acid bacteria with recognized health-conferring properties (12). A spore monoculture has the advantage of being readily produced, having a long shelf life, and, in the case of *B. subtilis*, being avirulent (11). *B. subtilis* has been studied and used as a feed additive to improve broiler performance (13, 14), modulate immune response (15, 16) and act as a prophylactic agent against bacterial diseases, by balancing gut microbiota (17, 18).

Some probiotics may be able to decrease the invasiveness of pathogens, which use inflammation to enhance their own colonization, by decreasing innate inflammatory responses, including macrophage activation phenotypes. Probiotics are also well documented to increase modulation of adaptive immunity (19). These findings suggest a specific immune interaction of each probiotic strain used, and its abilities to improve protection against certain pathogens, maintaining health and homeostasis through intestinal and systemic immunomodulation, in order to enhance animal performance and health.

The objective of this trial was to evaluate the ability of a probiotic composed of three different *B. subtilis* strains to reduce the invasiveness and gut colonization of the Brazilian SH UFPR1 strain, and its effects on performance, intestinal mucosa morphology, immune cells dynamics (macrophages, CD4+ and CD8+ cells) in liver, and gut microbiota in broiler chickens.

## Materials and Methods

### Animals and Experimental Design

The experiment was conducted at Center of Immune Response in Poultry at Federal University of Parana, Curitiba, Brazil, and was approved by the Ethical Committee of Agricultural Sector of Federal University of Parana under approval number: 037/2016.

Six, previously disinfected, BSL-2 rearing rooms were used. Each room contained four battery cages (replications) stacked vertically with sterilized litter, nipple drinkers, automatic temperature and lighting controls, all under a negative pressured air system.

A total of 288 one-day-old male Cobb 500^®^ broilers were distributed in a completely randomized block design (each block is a room) with six treatments of four replicates and 12 birds each where PRO was fed at 0, 250, or 500 g/ton of feed in either SH-inoculated or non-inoculated birds, as shown in Table 1. At the initiation of the trial, birds were allocated at in such a way that equal average initial body weight per cage was obtained. The trial was carried out from 1 to 21 days of age.

**Table 1:** Treatments description.

Treatments	*Salmonella* Heidelberg	Probiotic^a^ added (g/ton of feed)
	Challenge	
Control	No	0
PRO 250	No	250
PRO 500	No	500
SH Control	Yes	0
SH + PRO 250	Yes	250
SH + PRO 500	Yes	500

*^a^Live spores of *Bacillus subtilis* (PRO) strains (NP122, B2, and AM0904; Sporulin^®^, Novus International Inc.)*.

Aiming at minimizing the possibility of unexpected *Salmonella* contamination, the chickens used in this trial corresponded to the male line of a grandparent stock farm not vaccinated against any type of *Salmonella*.

### Product and Dosage

The probiotic (PRO) used in this trial is a feed additive manufactured with three isolated live spores of *B. subtilis* strains (NP122, B2 and AM0904; Sporulin^®^, Novus International Inc.). PRO was provided at three different levels: 0 g/ton (Control and SH Control groups), 250 g/ton (PRO 250 and SH + PRO 250), or 500 g/ton (PRO 500 and SH + PRO 500; Table 1). The recommended dosage by the manufacturer is 250 g/ton, which provides 10^6^ spores per g of feed.

### Feed Formulation and Mix

A balanced basal diet was offered in mash form and was formulated to provide nutrients at or above requirement levels (20). Corn and soybean meal were used as main ingredients and no antibiotics or growth promoters were added. The diet was designed for a unique feeding phase (Starter) and it was offered to broilers *ad libitum* from 1 to 21 days of age for all treatments.

The basal diet was sterilized by autoclave at 120°C for 15 min. After this process, PRO, amino acids, vitamin and mineral premix were added according to each treatment, and mixed for 10 min using a 50 kg “V” mixer. Batches were mixed in such an order to avoid interference among treatments. The PRO supplemented diets were mixed at last. The mixer was cleaned after each batch.

### *S. enterica* serovar Heidelberg

*Salmonella enterica* serovar Heidelberg (SH), strain UFPR1 sequences were submitted to the database NCBI/biosample identified as SAMN06560104, GenBank: CP020101. This pathogen was isolated from commercial broiler carcasses obtained from a broiler farm located in the south of Brazil. Samples from 20 livers and ceca were collected randomly from one-day-old chicks and tested negative for *Salmonella*. At 3 days of age, chicks from the SH Control, SH + PRO 250, and the SH + PRO 500 groups were orally challenged with 10^7^ CFU of SH per chick. At 7 and 21 days of age, 12 birds from the SH Control, SH + PRO 250, and the SH + PRO 500 cages were subjected to necropsy, while *Salmonella* sp. counts were quantified in liver and cecum samples. A pool of four ceca and four livers per treatment (Control, PRO 250, and PRO 500 birds) were also collected to evaluate the presence or absence of *Salmonella* sp. (qualitative analysis). In order to quantify typical colonies of *Salmonella* sp. (quantitative analysis), samples were processed using the modified methodology of Cox et al. (21). The abundance of *Salmonella* in ileum and cecum was also measured using metagenomic analysis.

### Performance

All chicks and feed were weighed weekly to evaluate feed intake (FI), body weight (BW), body weight gain (BWG), and feed conversion ratio (FC). All birds used for tissue sampling were weighed individually to estimate FC corrected for mortality. Mortality due to other causes rather than sampling procedures was not observed in this trial.

### Macrophages, CD4+ and CD8+ Cells Quantification by Immunohistochemistry

At 7 and 21 days of age, 12 birds per treatment (3 birds per replicate) were euthanized and the accessory lobe of their livers were collected. Immunohistochemistry was performed to obtain macrophage, CD4+ and CD8+ lymphocyte counts according to Lourenço et al. (22) using the rabbit macrophage clone RAM-11 Dako. The labeled cells were counted in an optical microscope (Nikon Eclipse E200, Sao Paulo, Brazil) with a 100× magnification objective. Five fields per bird, totalizing 25 microscopic fields per treatment of liver, were measured using only the hepatic parenchyma aiming at avoiding lymphoid aggregates.

### Evaluation of Intestinal Health—Histology by ISI (I See Inside Methodology)

At 7 and 21 days of age, 12 birds per treatment (3 birds per replicate) were euthanized, liver and ileum samples collected and further subjected to microscopic evaluation using the ISI Methodology (“I See Inside”; Pat. INPI-BR1020150036019) (23) as published by Kraieski et al. (24). Shortly, this methodology was developed based on a numeric score of histological alterations. For each alteration observed during microscopic analysis, an impact factor (IF) is defined according to its importance in affecting organ functional capacity based on previous knowledge of literature and background research (e.g., necrosis has the highest IF because the functional capacity of affected cells is totally lost). The IF ranges from 1 to 3, where 3 represents an IF of the greatest significance in terms of the organ function. In addition, the extent of each alteration (intensity or observed frequency compared to non-affected tissue) is evaluated per field (liver) or per villi (intestine) and scored ranging from 0 to 3. To reach the final ISI value, the IF of each alteration is multiplied by the respective score number, and the results of all alterations are summed.

### Genomic DNA Purification of Luminal Gut Microbiota and DNA Sequencing

The ileal (distal) and cecal luminal contents from 12 birds (3 birds per replicate) of the Control, SH Control, SH + PRO 250, and the SH + PRO 500 treatments were collected, frozen in liquid nitrogen and stored at −80°C until further analysis. Genomic DNA from each sample was purified using QIAamp Fast DNA Stool Mini Kit (QIAGEN, Hilden, Germany) according to the manufacturer, and then DNA quantification and quality were evaluated using the NanoVue Plus spectrophotometer (GE Healthcare, Marlborough, USA). Samples were diluted at 50 ng/μL and pooled using the same volume for each one (three samples were used to form one pool, resulting in four replicates per treatment). The pooled samples from ileum and cecum were used to amplify approximately 460 bp of the 16S ribosomal RNA by PCR using specific primers V3 and V4. The PCR products were used to build the metagenomics library for sequencing using MiSeq Reagent kit v3 (600 cycle) (Illumina Inc.). The sequencing of partial 16S ribosomal RNA was performed by next-generation sequencing method using Illumina MiSeq platform that produced thousands of 300 bp paired-end reads (2 × 300 bp) for each library. The full-length primer sequences to follow the protocol targeting this region are: 16S Amplicon PCR Forward Primer = 5′ TCGTCGGCAGCGTCAGATGTGTATAAGAGACAGCCTACGGGNGGCWGCAG and 16S Amplicon PCR Reverse Primer = 5′ GTCTCGTGGGCTCGGAGATGTGTATAAGAGACAGGACTACHVGGGTATCTAATCC.

### Processing of the Reads and Phylogenetic Analysis

The sequencing data were analyzed in the Bioinformatics Lab of the UNICAMP (www.lge.ibi.unicamp.br). The paired-end reads from each treatment were submitted to quality filtering and adapter trimming using Trim Galore software (http://www.bioinformatics.babraham.ac.uk/projects/trim_galore). The trimmed paired-end reads were merged into single reads using PEAR software (25). The single reads were then submitted to phylogenetic analysis and taxonomic assignments of the V3-V4 portion of the 16S rRNA gene using QIIME package (26) configured for constructing Operational Taxonomic Units (OTUs) with 97% of identity and assign taxonomy based on the Greengenes reference database (currently version 13_8). The full data sequence has been registered at NCBI BioProject and the information should be available at the following link: http://www.ncbi.nlm.nih.gov/bioproject/413291. The rarefaction curves were conducted to evaluate the coverage of OTUs.

### Diversity Analysis and Comparison among Treatments

Only taxonomic groups with abundance higher than 1% at the deepest level identified were submitted to cluster analysis. The clustering of different treatments was done using the Multiple Experiment Viewer software (27). Ecological indexes, such as diversity $H^{\prime }=-\sum_{i=1}^{s}p_{i}\text{ ln }p_{i}$; where *p_i_* is the proportion of characters belonging to the i*th* type of letter in the string of interest (28), richness and equitability $J=\frac{H^{\prime }}{H_{{\text{max}}^{"}}}$; where $H_{{\text{max}}^{\prime }}=\log_{b}S$, were calculated using the program R. For all ecologic indexes, all OTUs obtained were used except those that appeared only once.

### Statistical Analysis

Data were analyzed using the statistical software Statistix 9^®^. The microbiological data were evaluated by the Shapiro–Wilk normality test. The parametric data were subjected to analysis of variance (ANOVA) and Tukey’s test to establish differences among treatment means. The nonparametric data were submitted to the Kruskal–Wallis test at a 5% probability value. When presence or absence of *Salmonella* was assayed, the chi-square test was used to establish statistical differences. For performance, immunohistochemistry, and histology analysis, data were submitted to ANOVA using a 2 × 3 factorial design, once no difference for block were observed. Changes in the populations of individual bacteria were analyzed by ANOVA and Tukey’s test accordingly. For heat maps, only bacteria with abundance higher than 1% were used. A complete list of microorganisms identified are showed in Table S1 in Supplementary Material for ileum and Table S2 in Supplementary Material for cecum.

## Results

There was no interaction between SH and PRO birds for live performance and SH did not affect these parameters at any age period. The addition of PRO at 250 g/ton increased (*P* < 0.05) FI and BWG from 1 to 21 days compared to Control (Table 2).

**Table 2:** Mean ± SD of feed intake (FI) (g), body weight gain (BWG) (g), and feed conversion (FC) during the periods 1 to 7, 1 to 14, and 1 to 21 days of age.

	FI 1–7 (g)	FI 1–14 (g)	FI 1–21 (g)	BWG 1–7 (g)	BWG 1–14 (g)	BWG 1–21 (g)	FC 1–7	FC 1–14	FC 1–21
**Challenge**					**Main effects**				
Control	122.99 ± 8.54	468.06 ± 35.08	1079.7 ± 83.76	110.53 ± 2.49	390.83 ± 12.09	886.97 ± 27.03	1.112 ± 0.01	1.202 ± 0.02	1.220 ± 0.01
SH	116.99 ± 13.03	463.20 ± 35.56	1093.0 ± 62.82	107.20 ± 2.98	408.63 ± 14.76	916.90 ± 32.57	1.091 ± 0.01	1.142 ± 0.02	1.202 ± 0.03

**Probiotic**									
Control	114.79 ± 14.63	447.58 ± 49.59	**1035.2 ± 83.95^b^**	103.25 ± 3.86	374.65 ± 19.95	**839.11 ± 38.50^b^**	1.110 ± 0.02	1.204 ± 0.03	1.243 ± 0.03
250	127.45 ± 5.88	482.56 ± 18.51	**1131.3 ± 48.07^a^**	114.24 ± 1.85	427.26 ± 13.61	**965.98 ± 31.46^a^**	1.116 ± 0.01	1.137 ± 0.03	1.176 ± 0.02
500	116.98 ± 7.53	466.56 ± 17.51	**1094.5 ± 43.77^a,b^**	108.89 ± 3.13	398.22 ± 8.29	**902.68 ± 23.25^a,b^**	1.075 ± 0.02	1.173 ± 0.01	1.214 ± 0.01

**Probabilities**									
Challenge (P_1_)	0.204	0.763	0.659	0.374	0.350	0.483	0.496	0.098	0.655
Probiotic (P_2_)	0.058	0.163	**0.031**	0.071	0.062	**0.040**	0.411	0.229	0.278
Interaction (P_1_ × P_2_)	0.639	0.755	0.865	0.743	0.259	0.410	0.787	0.061	0.336

*^a,b^Different letters in the same column indicate significant differences at P < 0.05 at Tukey’s test*.

*Shading was used to distinguish columns from FI, BWG,FC*.

*Bold values were used to distinguish statistical differences*.

**Figure 1:**
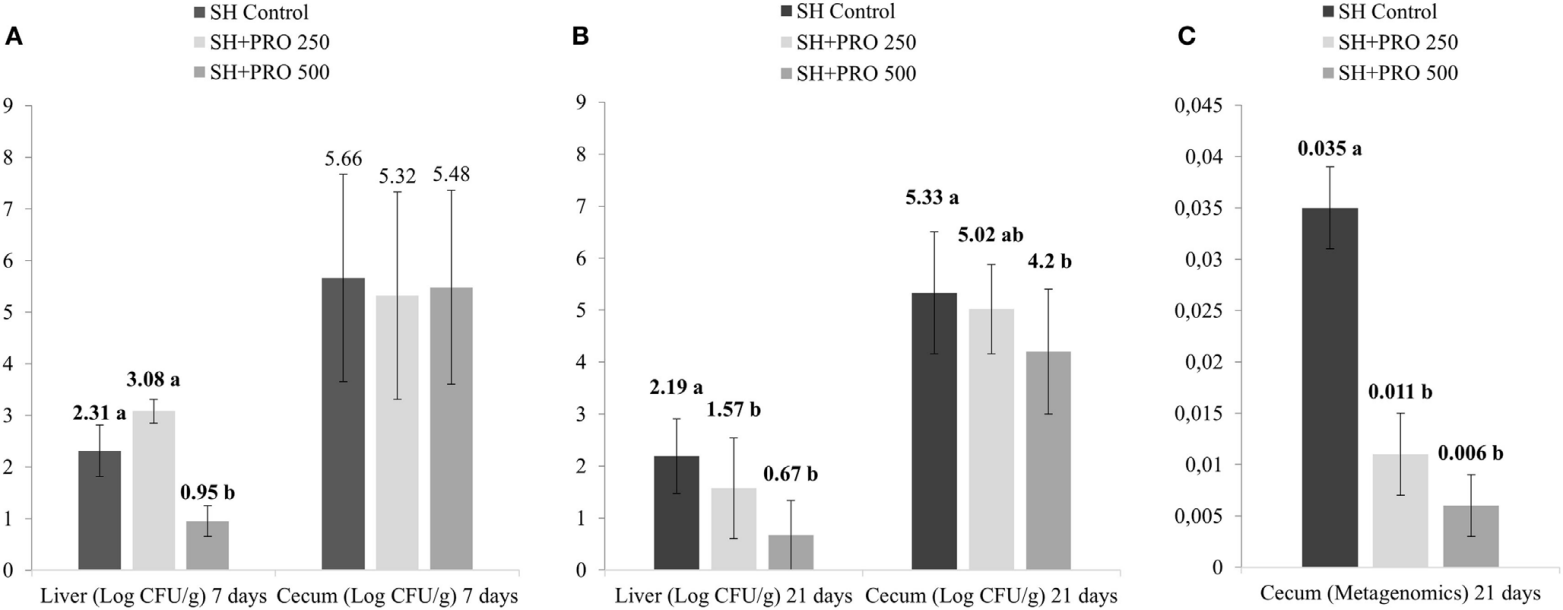
*Salmonella* sp. quantification. **(A)** *Salmonella* sp counts (Log CFU/g) in liver and cecum at 7 days of age (4 days after inoculation) in treatments SH Control, SH + PRO 250, and SH + PRO 500 according to adapted methodology by Cox et al. (21). **(B)** *Salmonella* sp. counts (Log CFU/g) in liver and cecum at 21 days of age in treatments SH Control, SH + PRO 250, and SH + PRO 500 according to adapted methodology by Cox et al. (21). **(C)** Relative abundance using metagenomics analysis in ceca at 21 days of age in treatments SH Control, SH + PRO 250, and SH + PRO 500. Non-challenged groups (Control, PRO 250, and PRO 500) were negative for *Salmonella* in both methodologies. Different letters indicate significant differences at *P* < 0.05 at Kruskal–Wallis.

As expected, the non-challenged Control, PRO 250, and PRO 500 groups tested negative for *Salmonella* therefore data were analyzed using the SH challenged treatments only as a completely randomized design. In liver, the SH + PRO 500 chicks had reduced SH counts (*P* < 0.01) compared to the SH Control birds at 7 days (Figure 1A), whereas both the SH + PRO 250 and the 500 birds had reduced SH counts at 21 days (*P* < 0.01) compared to the SH Control group (Figure 1B). In ceca, only the SH + PRO 500 group had reduced (*P* < 0.05) *Salmonella* counts (Figure 1B) using the bacteriological quantification (21). However, the PRO when fed at either dose significantly reduced *Salmonella* frequencies in cecum according to the more refined metagenomic analysis (Figure 1C) at 21 days of age.

Liver histologic alterations by ISI and immunohistochemistry analysis are summarized in Tables 3 and 4, respectively. No differences in ISI scores in liver were found among treatments in non-challenged birds at 7 days. Still, immunohistochemistry analysis revealed that the PRO fed at 500 g/ton reduced macrophages and CD4+ cells recruitment in the liver of those chickens compared to the Controls (*P* < 0.05).

**Table 3:** Mean ± SD of histological alterations (ISI) in liver (score per field) and ileum (score per villi) at 7 and 21 days of age.

	Liver		Ileum	
	7 days	21 days	7 days	21 days
**Challenge**				
Control	**23.49 ± 6.53^a^**	**12.26 ± 5.78^b^**	5.29 ± 4.39	9.99 ± 4.55
SH	**20.25 ± 7.13^b^**	**20.63 ± 6.61^a^**	4.56 ± 4.54	10.42 ± 3.54

**Probiotic**				
Control	**24.09 ± 5.49^a^**	**16.84 ± 6.74^b^**	**4.36 ± 4.32^b^**	**9.11 ± 4.16^b^**
250	**20.82 ± 7.63^b^**	**17.48 ± 7.41^b^**	**4.42 ± 4.24^b^**	**11.52 ± 3.95^a^**
500	**19.08 ± 7.09^b^**	**19.19 ± 7.06^a^**	**5.71 ± 4.84^a^**	**10.21 ± 3.20^b^**

**Interaction**				
Control	**23.70 ± 5.82^a^**	**9.93 ± 5.25^c^**	5.01 ± 0.57	**10.50 ± 5.22^a,b^**
PRO 250	**23.10 ± 7.27^a^**	**11.40 ± 5.28^c^**	4.11 ± 0.57	**10.47 ± 4.94^a,b^**
PRO 500	**23.67 ± 6.52^a^**	**15.45 ± 5.42^b^**	6.75 ± 0.57	**9.00 ± 3.13^b,c^**
SH Control	**24.28 ± 5.34^a^**	**20.31 ± 6.39^a^**	4.03 ± 0.40	**8.42 ± 3.33^c^**
SH + PRO 250	**19.67 ± 7.58^b^**	**20.52 ± 6.38^a^**	4.57 ± 0.40	**12.04 ± 3.24^a^**
SH + PRO 500	**16.79 ± 6.23^c^**	**21.06 ± 7.06^a^**	5.13 ± 0.42	**10.79 ± 3.08^a^**

**Probabilities**				
Challenge (P_1_)	**<0.001**	**<0.001**	0.081	0.204
Probiotic (P_2_)	**<0.001**	**<0.001**	**0.002**	**<0.001**
Interaction (P_1_ × P_2_)	**<0.001**	**<0.001**	0.106	**<0.001**

*^a,b,c^Different letters in the same column indicate significant differences at P < 0.05 at Kruskal–Wallis test*.

*Bold values were used to distinguish statistical differences*.

**Table 4:** Mean ± standard error of macrophages, CD4+ and CD8+ cells quantification by immunohistochemistry in liver (cells per field at 100× of magnification) at 7 and 21 days of age.

	Macrophages		CD4+		CD8+	
	7 days	21 days	7 days	21 days	7 days	21 days
**Challenge**						
Control	19.25 ± 1.09	10.88 ± 0.66	3.48 ± 0.26	4.35 ± 0.28	**4.63 ± 0.31^b^**	4.50 ± 0.29
SH	23.96 ± 0.33	11.78 ± 0.61	3.53 ± 0.10	4.12 ± 0.25	**5.30 ± 0.13^a^**	4.78 ± 0.25

**Probiotic**						
Control	**20.97 ± 0.95^b^**	**8.32 ± 0.65^b^**	**4.00 ± 0.22^a^**	3.50 ± 0.28	**5.53 ± 0.33^a^**	3.92 ± 0.28
250	**25.35 ± 0.47^a^**	**13.21 ± 0.79^a^**	**3.63 ± 0.16^a^**	4.85 ± 0.35	**5.05 ± 0.15^a,b^**	5.76 ± 0.34
500	**20.87 ± 1.01^c^**	**12.91 ± 0.80^a^**	**2.92 ± 0.17^b^**	4.25 ± 0.35	**4.67 ± 0.20^b^**	4.38 ± 0.33

**Interaction**			**Interactions**			
Control	**20.15 ± 1.35^b^**	7.70 ± 1.30	**4.40 ± 0.57^a^**	**5.15 ± 0.56^a^**	**4.65 ± 0.79^a,b^**	4.1 ± 0.56
PRO 250	**26.80 ± 1.11^a^**	11.4 ± 1.3	**4.15 ± 0.24^a^**	**4.25 ± 0.43^a,b^**	**5.30 ± 0.30^a,b^**	5.50 ± 0.56
PRO 500	**10.80 ± 1.13^c^**	13.55 ± 1.3	**1.90 ± 0.22^b^**	**3.65 ± 0.41^a,b^**	**3.95 ± 0.40^b^**	3.90 ± 0.56
SH Control	**21.37 ± 0.72^b^**	8.62 ± 0.92	**3.80 ± 0.16^a^**	**2.67 ± 0.22^b^**	**5.97 ± 0.28^a^**	3.82 ± 0.39
SH + PRO 250	**24.62 ± 0.41^a^**	14.12 ± 0.92	**3.37 ± 0.20^a^**	**5.15 ± 0.47^a^**	**4.92 ± 0.17^a,b^**	5.90 ± 0.39
SH + PRO 500	**25.90 ± 0.26^a^**	12.6 ± 0.92	**3.42 ± 0.18^a^**	**4.55 ± 0.49^a^**	**5.02 ± 0.21^a,b^**	4.65 ± 0.39

**Probabilities**						
Challenge (P_1_)	**<0.001**	0.332	0.817	0.567	**0.020**	0.475
Probiotic (P_2_)	**<0.001**	**<0.001**	**<0.001**	0.233	**0.050**	**0.001**
Interaction (P_1_ × P_2_)	**<0.001**	0.271	**<0.001**	**0.001**	**0.035**	0.576

*^a,b,c^Different letters in the same column indicate significant differences at P < 0.05 at Kruskal–Wallis test*.

*Bold values were used to distinguish statistical differences*.

The challenged birds fed the PRO had livers with lower histological alteration scores compared to the SH Control group (*P* < 0.01) at 7 days of age. A reduction on hydropic degeneration and necrosis of liver parenchyma were associated with those observations. In addition, higher macrophage counts in liver were found in both the SH + PRO 250 and the 500 groups compared to the SH Control (Table 4). This could be related to the SH reduction in this organ (at least for the PRO when fed at 500 g/ton). The opposite was observed in non-challenged birds when the PRO 500 chicks exhibited reduced (*P* < 0.01) macrophages and CD4+ cells in liver parenchyma.

At 21 days of age, the PRO 500 birds had increased ISI liver scores compared to the Control and the PRO 250 groups in non-challenged birds (Table 3). No differences were found in the SH-challenged broilers on this parameter. Still, increased CD4+ cells counts were observed in both the SH + PRO 250 and the SH + PRO 500 groups compared to the SH Control birds (Table 4). The macrophage counts were higher in liver at 21 days of age regardless of the SH challenge.

**Figure 2:**
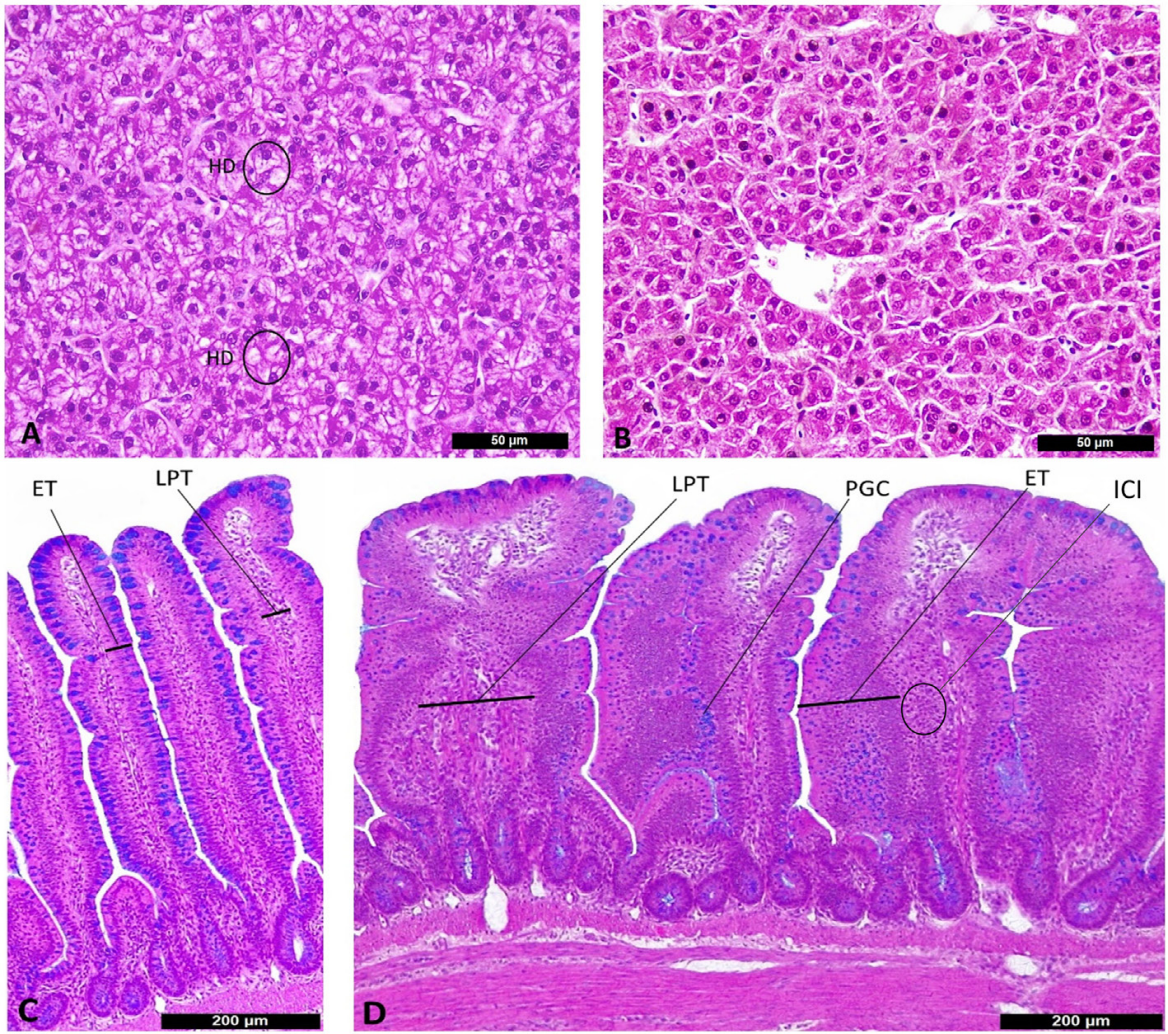
Histological alterations in liver **(A,B)** and ileum **(C,D)** according to I See Inside (ISI) scoring methodology (100×). **(A)** Liver from SH Control, presenting score 3 of hydropic degeneration (HD) at 7 days of age. **(B)** Liver from SH + PRO 500, normal hepatocytes at 7 days of age. **(C)** Ileum from SH Control, villi with scores zero for epithelial thickness (ET) and lamina propria thickness (LPT) at 21 days of age. **(D)** Ileum from SH + PRO 250 with score 2 for epithelial thickness (ET), score 2 for proliferation of goblet cells (PGCs) and score 2 for LPT with inflammatory cells infiltration (ICI) at 21 days of age.

Birds fed PRO at 500 g/ton had higher ISI scores in ileum at 7 days of age (Table 3). The main alterations observed in challenged birds were an increase in lamina propria thickness, epithelial thickness and proliferation of goblet cells (*P* < 0.05). At 21 days of age, a significant interaction for ileal ISI scores was found, where both the SH + PRO 250 and the SH + PRO 500 groups presented higher ISI scores than the SH Control, while no significant differences were observed in non-challenged birds (Table 3). The main histologic alterations found in the PRO 500 g/ton group at that age were also observed at 7 days (Figures 2C,D).

**Figure 3:**
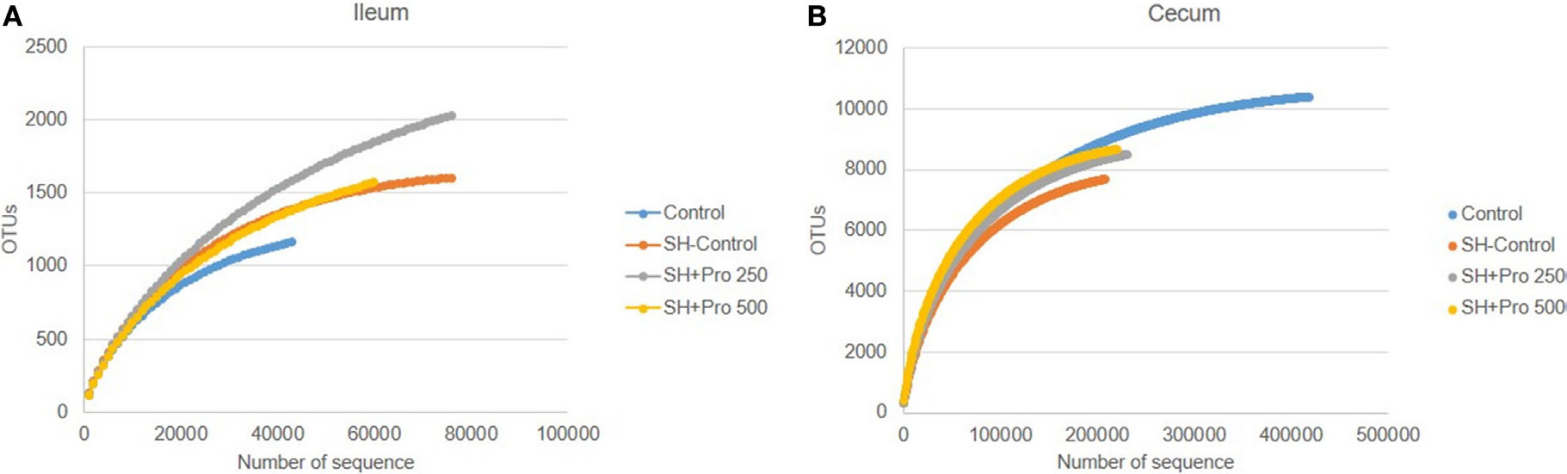
Rarefaction plot from ileal **(A)** and cecal **(B)** microbiota of groups Control, SH Control, SH + PRO 250, and SH + PRO 500. *P < 0.05. **P = 0.08. Rarefaction analysis suggested that the number of sequences from all experimental samples were enough to uncover major Operational Taxonomic Units (OTUs).

**Figure 4:**
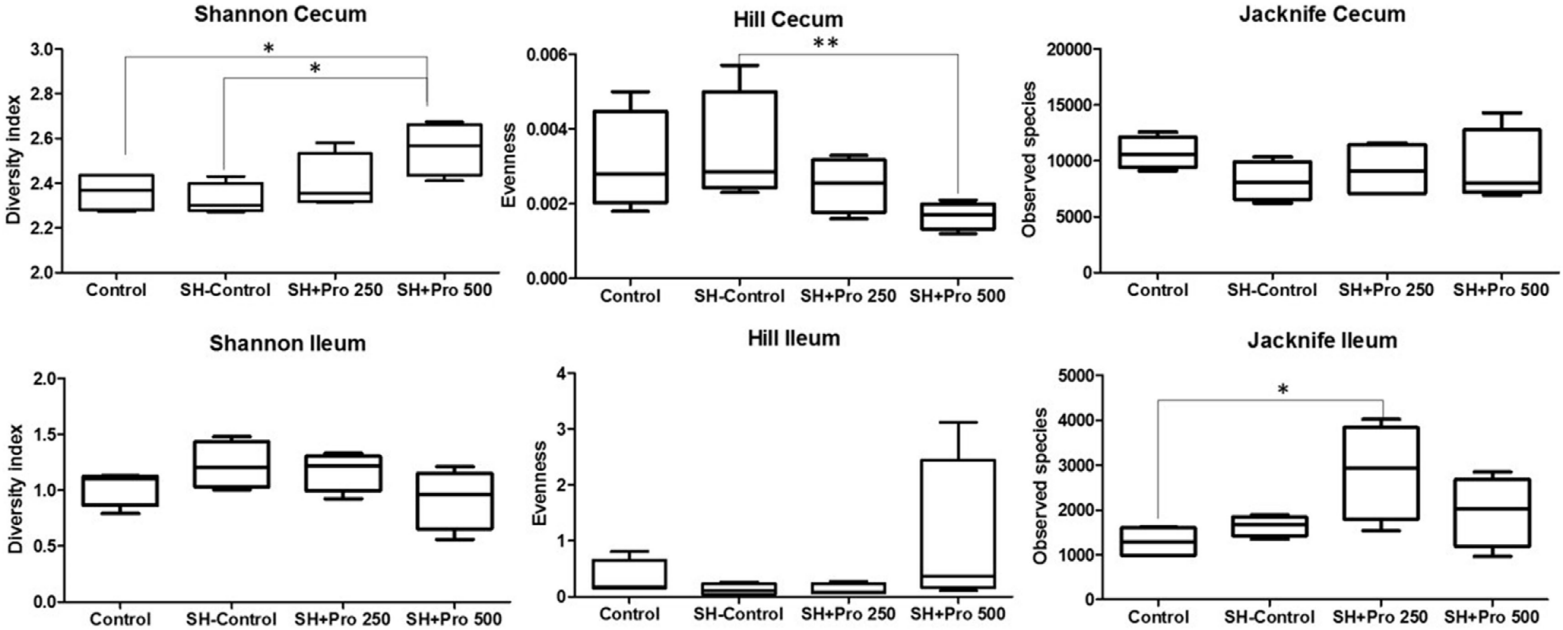
Ileal and cecal diversity (Shannon–Wiener), evenness (Hill), and richness (JackkNife) index of groups Control, SH Control, SH + PRO 250, and SH + PRO 500. *p < 0.05. **p = 0.08.

The metagenomic analysis of gut microbiota revealed an average of 411.360 and 157.658 reads per sample of cecum and ileum, respectively. Based on 97% species similarity, an average of 9.330 and 1.942 operational taxonomic units (OTUs) were obtained in cecum and ileum, respectively. The rarefaction curves suggested that in all treatments enough sequence reads per sample were collected, showing that sampling has been exhaustively sequenced and was enough to uncover major OTUs (Figure 3). The diversity index by Shannon–Wiener revealed that cecal microbial composition of the SH + PRO 500 group was significantly more diverse compared to the Control and the SH Control groups. The SH + PRO 250 birds had significant (*P* < 0.05) higher richness (JackkNife test) in ileal microbiota compared to the Control group, while evenness test (Hill) revealed that the SH + PRO 500 birds have lower species evenness in the cecum compared to the SH Control group (Figure 4).

**Figure 5:**
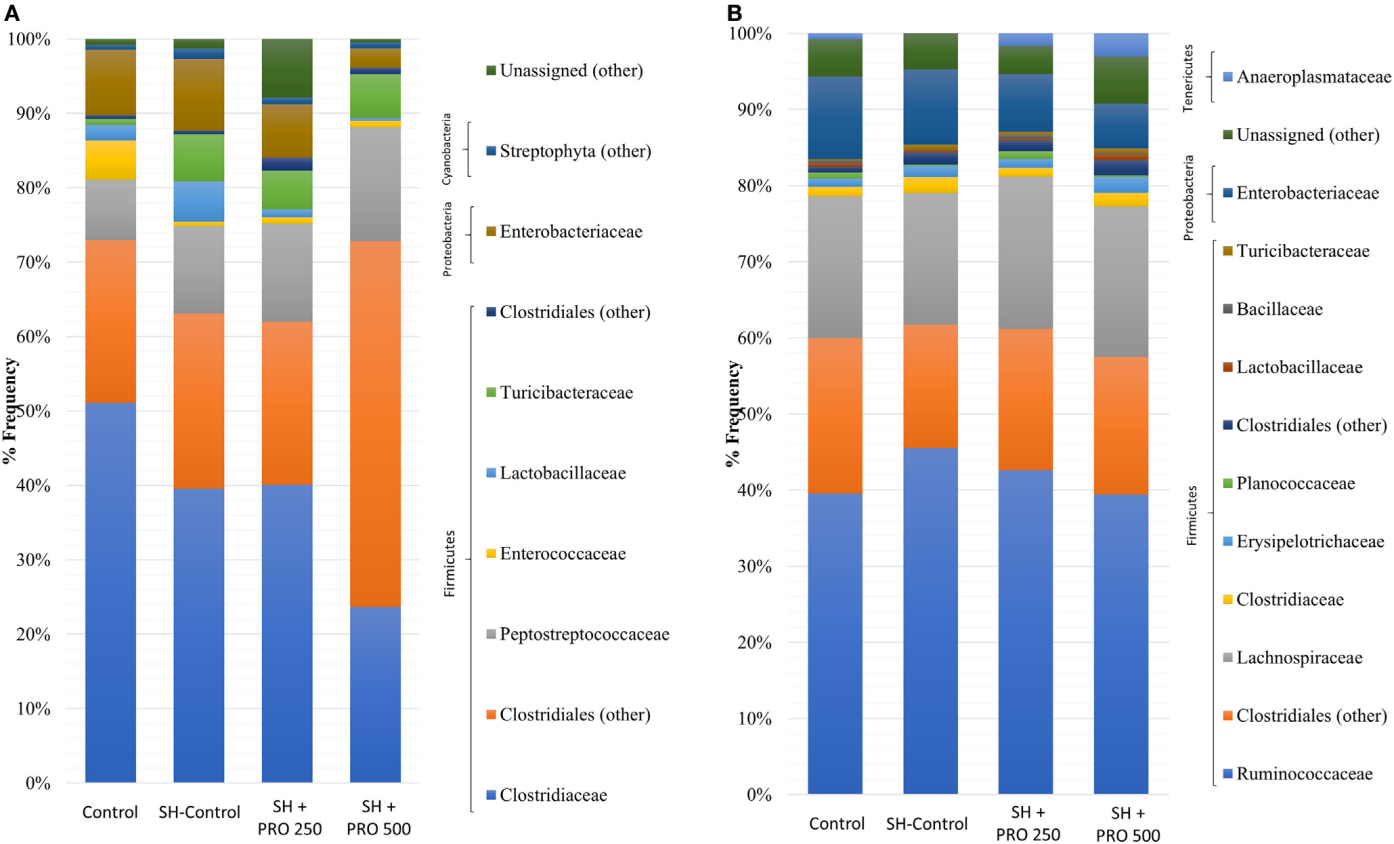
Relative abundance of bacteria population in ileal microbiota **(A)** and cecal microbiota **(B)** of groups Control, SH Control, SH + PRO 250, and SH + PRO 500 at 21 days of age, analyzed by sequencing using Illumina MiSeq System.

**Figure 6:**
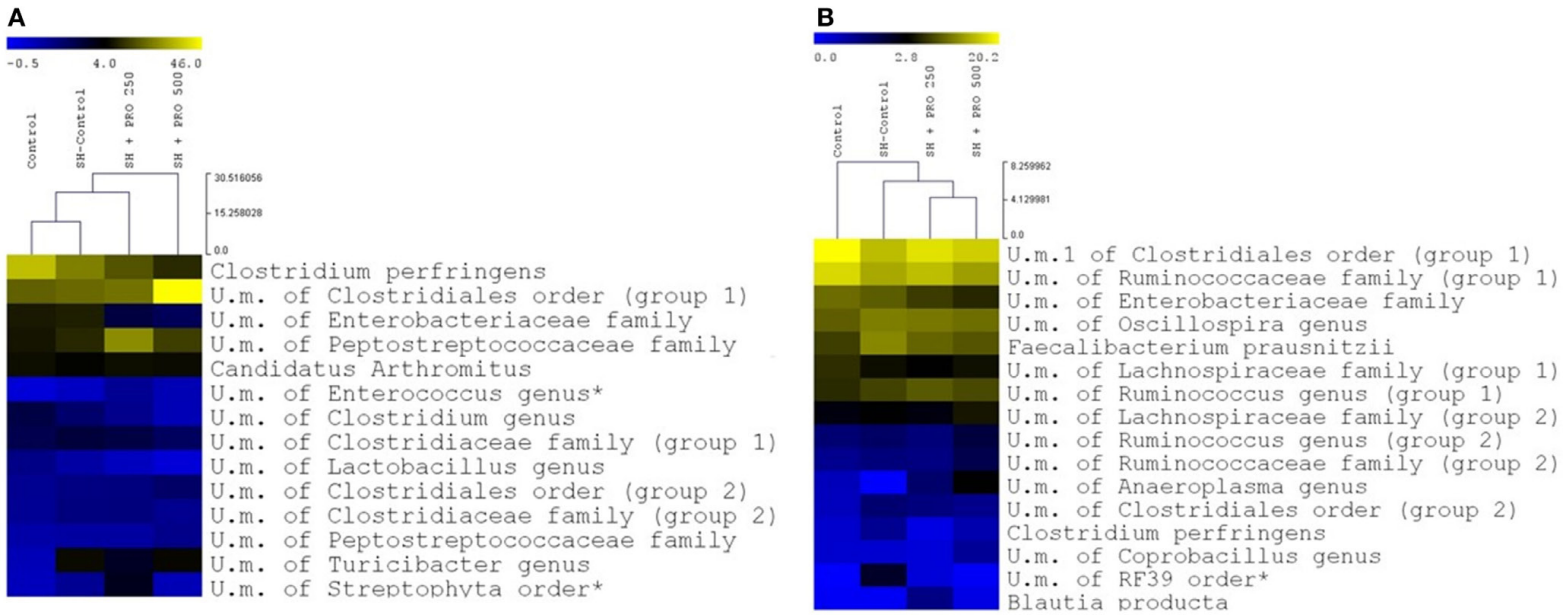
Relative abundance of distinct groups at the deepest level identified in ileum **(A)** and cecum **(B)** of groups Control, SH Control, SH + PRO 250, and SH + PRO 500. A yellower color depicts a greater bacterial abundance to up to 46.2% in ileum **(A)** abundance and up to 20.2% in cecum **(B)** abundance. Groups with abundance less than 1% were not considered. U.m., unidentified members. * Indicate significant differences at *P* < 0.05.

The family profiles of the corresponding ileal microbial populations are shown in Figure 5A. As expected, the data on microbiota presented high coefficients of variation addressing the difficulties in establishing statistical differences. The Clostridiaceae family (mostly represented by *Clostridium perfringens*) presented numerically lower abundance in the SH + PRO 500 chickens. *C. perfringens* were detected in high quantity in ileum because the samples were collected in the distal section. The unidentified members of Clostridiales order (group 1) revealed higher numerical abundance in the SH + PRO 500 broilers as opposed to other groups. The unidentified members of *Enterococcus* genus (phylum Firmicutes) and members of Peptostreptococcaceae family (group 1; class Clostridia) were significantly higher (*P* < 0.05) in the SH + PRO 250 chickens compared to the Control ones (Figure 6A). Another significant difference in ileum (*P* < 0.05) is related to unidentified members of Streptophyta order, within the Cyanobacteria phylum. This bacterium was more abundant in the SH + PRO 250 group compared to the Control and the SH + PRO 500 treatments (Figure 6A).

In cecal microbiota, the majority of Clostridiales detected fall primarily into Ruminococcaceae, Lachnospiraceae and Clostridiaceae families (Figure 5B). An unidentified member of RF39 order (phylum Tenericutes, class Mollicutes) presented a statistical difference (*P* = 0.041) between the Control and the SH Control cages (Figure 6B). The abundance of *Salmonella sp* in ceca was lower than 1% (i.e., up to 0.035%) been significantly lower in broilers fed PRO at both dosages comparing to the Control and the SH Control birds (Figure 1C; *P* < 0.05).

## Discussion

No loss in performance resulted from challenging birds with SH at any time. This agrees with previous studies in our laboratory which showed that not all *Salmonella* influence the performance of broilers (29). As the current trial was not primarily designed to test performance, the experimental layout had low statistical power to detect differences in parameters such as intake and weight gain. Still, a significant improvement in performance resulted from feeding PRO at 250 g/ton. This has also been observed by other workers when feeding some *B. subtilis* strains to broilers (13, 14, 30).

It is worth noticing that the resulting abundance of *Salmonella* in cecum was relatively low (up to 0.035% for the SH Control group) compared to other bacterial groups (Figures 5 and 6); and that it was not detected in the ileum of chickens even in those orally challenged with SH, confirming the low affinity of *Salmonella* for that organ. Still, Feeding PRO at 500 g/ton reduced *Salmonella* counts in both liver and cecum by the end of the trial. In the latter organ, metagenomics showed that both dosages were equally effective in reducing *Salmonella* abundance.

Other studies (17) have also shown that adding *B. subtilis* spores in the diet could reduce SH colonization at 42 days of age by up to 58%. The most commented mechanism been competitive exclusion by which *B. subtilis* bacteria occupy adhesion locations of the membranes of enterocytes, goblet and enteroendocrine cells regularly used by *Salmonella*, therefore preventing it from establishing itself in the gut (31). An agonist effect caused by the secretion of substances by *B. subtilis*, such as bacteriocins, organic acids, and hydrogen peroxide, can also inhibit the growth and development of pathogenic bacteria. Likewise, some strains of *B. subtilis* are known to favor the growth of lactic acid-producing bacteria (12) with a subsequent acidification of the intestinal environment (32). These effects could modulate the host’s microbial populations and the intestinal immune response potentially reducing the frequency of *Salmonella* in the gut and its capacity to migrate from the intestinal lumen into other organs. These are in agreement with the observations in the current trial.

Feeding PRO may help to reduce some deleterious alterations in liver parenchyma caused by SH. Hydropic degeneration is an intracytoplasmic fluid accumulation, secondary to disturbance of cell membrane integrity causing vacuolation of hepatocytes (Figures 2A,B). One of the causes is bacterial infections with differing lobular localization and may be a precursor to hepatocyte necrosis (33). Also, the interesting transport of immune cells of PRO in liver was reported by other study (34) where probiotic bacteria reduced monocyte and macrophage recruitment to the intestines and spleen compared to control animals. Probiotics may ameliorate proinflammatory immune cell recruitment to systemic lymphoid tissues such as liver and other organs. This could save metabolic energy and have positive effect on performance, which in the present trial was observed in broilers fed PRO at 250 g/ton of feed. This performance improvement was not observed when feeding PRO at the highest dose, however, this group of birds showed a significant reduction in *Salmonella* infection when challenged with SH, recruited macrophages to eliminate bacteria by phagocytosis, secreted cytokines to modulate immunity and presented antigens to helper T cells (35).

The relationship between chicken macrophages and *Salmonella*, as well as intracellular survival of *Salmonella* in chicken macrophages, remains poorly understood. According to Van Immerseel et al. (36), the encounter between specialized epithelial cells and microorganisms quickly stimulates the release of proinflammatory chemokines that attract innate immune cells (i.e., granulocytes and macrophages), which are able to trigger a wide range of new immune responses such as the emergence of T helper lymphocytes (CD4+ cells). An early increase in CD4+ and CD8+ cells has been reported in chickens fed probiotics (37–39). In some cases, *Salmonella* cells invade and multiply within the macrophages (40–42) and widely distribute themselves in the lymphoid and nonlymphoid tissues, facilitating their spreading to various organs of the host.

In this study, histology observations in ileum seemed atypical as reported in other *Salmonella* trials (37, 38) suggesting a considerable variation on ileal morphology when *Salmonella* is present. This variation in ileum histology could be associated with the fact that *Salmonella* has the cecum as target tissue.

Some alterations were observed on ileum histology due to PRO activity such that lamina propria and epithelial thickness increased along with goblet cells proliferation. Probiotics exert a range of effects on mucosal barrier function and on responses of the underlying immune tissue of the gut associated with lymphoid tissue (19). This barrier function is enforced by the ability of probiotics to influence mucin expression and mucus secretion of goblet cells. It is likely that the probiotic-mediated modulation of mucin expression is a host’s strategy to allow beneficial microbes to colonize the gut (43). Furthermore, mucins may exert prebiotic-type effects as carbohydrate content can account for 90% of their weight (44). Muniz et al. (37) observed similar effects when four different probiotics increased the proliferation of goblet cells in ileum. The association of probiotics with epithelial cells might be sufficient to trigger signaling cascades at epithelium level and activate underlying immune cells in lamina propria (45). Probiotics may increase epithelial and lamina propria thickness, characterized by cell proliferation and inflammatory cells infiltration, respectively (Figure 2D), describing a mucosal wound repair (46). In a recent publication, Kraieski et al. (24) observed a positive correlation between ileal epithelial thickness and goblet cells proliferation with BWG, and a negative correlation with FC at 21 days of age. In the present experiment, PRO fed at 250/ton improved BWG while the SH + PRO 250 group presented higher ileal ISI than the SH Control birds at 21 days along with increased epithelial thickness, goblet cells proliferation and lamina propria thickness.

The metagenomics analysis also showed a significant increase in *Bacillus* genus abundance in the ileum of birds fed PRO going from 0.004 ± 0.002% for the Control group to 0.019 ± 0.004% for the SH + PRO 500 animals (Table S1 in Supplementary Material). That could be due to the presence of *Bacilli* from PRO in that organ itself or, could have been the result of gut microbial changes in *Bacilli* populations not necessarily of PRO origin, since the *Bacillus* genus is commonly found in the ileal microbiota of broilers.

The diversity index by Shannon–Wiener revealed that cecal microbial composition of the SH + PRO 500 group was significantly more diverse compared to the Control and the SH Control groups (Figure 4). Pereira (47) detected less diversity in chickens fed with *B. subtilis* spores. However, it has been reported that the use of probiotics can increase the intestinal microbiota diversity in different organisms (48, 49). Diversity is a combination of richness and evenness. Increasing the diversity tends to suggest more stable ecosystems with more connections within them, even though statistical differences in performance were not observed in the SH + PRO 500 treatment.

In general, the most abundant phylum in the chicken intestinal microbiota is Firmicutes followed by two minor phyla, Proteobacteria and Bacteroidetes. In addition, members of phylum Actinobacteria, Tenericutes (50), Cyanobacteria, and Fusobacteria (51) can be found in very low abundance. In the present study, Firmicutes was the most predominant phylum found in ileum and cecum in all groups. Proteobacteria, Cyanobacteria (ileum), and Tenericutes (cecum) were also observed but showing lower abundance (Figures 5A,B).

*Enterococcus* (phylum Firmicutes) is a large genus of lactic acid bacteria, commensals of animal and human gut (52). In ileum, this genus was significantly higher (*P* < 0.05) in the SH + PRO 250 rather than in the Control group (Figure 6A). Many enterococci species such as *E. faecium* produce bacteriocins which have been associated with growth inhibition of food-borne pathogens in the gut (53). It might be possible that increases in the relative abundance of above mentioned commensals in probiotic treated chickens reduced *Salmonella* colonization or simply contributed to intestinal health. Members of Peptostreptococcaceae family (class Clostridia) seemed to be more abundant in the SH + PRO 250 broilers compared to the Control group (*p*=0.06). The Peptostreptococcaceae was isolated from various environments including clinical human and animal samples, manure, soil, marine and terrestrial sediments, and deep-sea hydrothermal vents. High percentage of Peptostreptococcaceae was found in ileal samples from conventional broiler chickens at 7 and 41 days of age, assuming that this family might be considered a commensal bacteria group (54). Another significant difference in ileum (*P* < 0.05) is related to unidentified members of Streptophyta order, within the Cyanobacteria phylum, that could be attributed to chloroplasts, non-photosynthetic bacteria commonly found in the animal gut (55). This bacterium was more abundant in the SH + PRO 250 group compared to the Control and the SH + PRO 500 treatments (Figure 6A).

An unidentified member of RF39 order (phylum Tenericutes, class Mollicutes) was more abundant than in Control when SH was present while feeding PRO could reduce it numerically in cecal microbiota (Figure 6B). In past studies, it was reported that Mollicutes were enriched in birds affected by necrotic enteritis disease and this could possibly be associated with intestinal disorders for chickens (56). However, Perez-Brocal et al. (57) observed that humans with Crohn’s disease (inflammatory bowel disease) showed lower abundance of bacteria from RF39 order compared to the Control group. Goodrich et al. (58) observed an increase of RF39 order in lean body mass adults, compared to obese individuals. Besides the lack of information in literature, it is not possible to assume correlations with those data once the genus from RF39 order was unidentified in the current experiment.

## Conclusion

A probiotic composed by three strains of *B. subtilis* improved animal performance when fed at 250 g/ton and reduced *Salmonella* colonization in liver and cecum at 250 and 500 g/ton when birds were orally challenged with SH strain UFPR1. The mobilization of immune cells in liver can be a relevant mode of action of PRO in birds challenged with SH. PRO can promote important histologic alterations related to activation of defense response and gut absorption. In addition, the supplementation of PRO increased the diversity of cecal microbiota, which suggests a more stable ecosystem, and increased some commensal bacterial groups in ileum, some of which are lactic-acid producing organisms.

## Ethics Statement

The experiment was approved by the Ethical Committee of Agricultural Sector of Federal University of Parana under approval number: 037/2016.

## Author Contributions

RH: *in vivo* trial, analysis of the data, microbiology, immunohistochemistry, and performance analysis; drafting and revising it critically and final approval of the version. ML: *in vivo* trial, microbiology analysis. AK: *in vivo* trial, statistical analysis. RA: microbiome analysis orientation, development of *B. subtilis*. RG-E: statistical analysis, development of *B. subtilis*. EL: microbiome analysis and interpretation of results. AC: microbiome analysis and interpretation of results. MC: microbiome analysis and interpretation of results. PM: microbiome analysis and interpretation of results. ES: orientation of the all experiment; drafting and revising it critically and final approval of the version.

## Conflict of Interest Statement

We would like declare that the study was supported by Novus International Inc. and the authors RA and RG-Esquerra were employees to Novus International Inc. All other authors have no competing interests to declare.
